# Detection, Control, Risk Assessment, and Prevention of Foodborne Microorganisms

**DOI:** 10.3390/foods13101551

**Published:** 2024-05-16

**Authors:** Arícia Possas, Fernando Pérez-Rodríguez

**Affiliations:** Department of Food Science and Technology, UIC Zoonosis y Enfermedades Emergentes ENZOEM, CeiA3, Universidad de Córdoba, 14014 Córdoba, Spain; b42perof@uco.es

Despite significant efforts from government and industry, enteric foodborne diseases continue to pose a substantial public health challenge worldwide. In the European Union, the number of deaths resulting from foodborne outbreaks in 2022 reached the highest level recorded since 2012 [1]. *Listeria monocytogenes* was identified as the primary cause of these deaths, followed by *Salmonella* spp. [1]. Furthermore, recent shifts in consumer behavior, the globalization of commerce, advancements in food processing technologies, and climate change have contributed to the emergence and re-emergence of foodborne diseases [2,3]. 

In response to these challenges, this Special Issue sought to gather original research that addresses these critical issues. The fifteen articles featured in this Special Issue cover a wide range of topics, from the development of advanced detection methods to the implementation of risk assessment frameworks using computational tools. The featured articles highlight the importance of interdisciplinary collaboration and the integration of cutting-edge technologies to advance our understanding of foodborne microorganisms and improve food safety practices. Moreover, the articles included herein offer valuable knowledge on the complex dynamics of foodborne disease emergence and provide practical solutions for microbial detection, prevention, and control.

In line with the goal of improving the detection of foodborne pathogens, Luo et al. (contribution 1) introduced a microfluidic chip integrating loop-mediated isothermal amplification and CRISPR/Cas12a systems for detecting *Salmonella*, addressing issues of aerosol pollution in DNA amplification. This innovative chip facilitates amplification at 65 °C for 20 min, followed by fluorescent signal production at 43 °C for 30 min, achieving a detection sensitivity of 118 pg/µL with 100% accuracy. Application of the microfluidic chip in salmon and chicken samples spiked with *Salmonella* showed stable detection capabilities. 

Furthermore, through their study, Niu et al. (contribution 2) made advancements in the detection of antimicrobial resistance mechanisms against quinolone and fluoroquinolone in foodborne pathogens by developing stable plasmid DNA reference materials. DNA fragments of 11 target genes were successfully synthesized, inserted into plasmid vectors, and transferred into recipient cells. Genetic stability, limit of detection, homogeneity, and storage stability were evaluated. All target DNA remained stable and detectable during subculturing, whereas plasmid DNA remained detectable after storage at various temperatures for different durations without mutations occurring. The materials developed in the study meet standard requirements and can be effectively used to detect resistance mechanisms in foodborne pathogens.

This Special Issue also presents studies on novel biopreservation strategies for controlling foodborne pathogens. Resendiz-Moctezuma et al. (contribution 3) screened the antimicrobial potential of organic acids and essential oils (EOs) as antimicrobials against *Salmonella* Typhimurium in pork loin. Their findings revealed significant reductions in the pathogen’s prevalence caused by lactic acid, formic acid, cumin, peppermint, and spearmint, although no interactions between these antimicrobial candidates were found in pork loin. Similarly, Gál et al. (contribution 4) investigated the effectiveness of sage EO and heat in inactivating *L. monocytogenes* in sous vide processed beef tenderloin. The study samples were cooked sous vide at different temperatures, and bacterial counts were assessed over 12 days. Both *L. monocytogenes* and coliform bacteria levels increased over time, with *Pseudomonas fragi* and *L. monocytogenes* being the most common isolated organisms. Notably, the addition of sage EO showed promise in ensuring the safety of sous vide beef tenderloin.

In addition to the use of strategies for reducing microbial loads in different types of food, other strategies for preventing microbial contamination and shedding have also been assessed. This is illustrated in the study by Jiménez et al. (contribution 5) on the effectiveness of physically removing lymph nodes from pork products prior to grinding in mitigating *Salmonella* and reducing indicator organisms in the final ground products. Three treatment groups were assigned in a commercial pork processing facility, with varying levels of lymph node removal. The results of their study showed a significant reduction in the presence of *Salmonella* and indicator organisms when topical and internal lymph nodes were removed before grinding. Their findings underscore the importance of implementing lymph node removal strategies to prevent contamination in pork products that undergo further processing.

The studies by Pasquali et al. (contribution 6) and Wiatrowski et al. (contribution 7) shed light on the crucial role of hygiene in ensuring food safety in different food processing environments. Pasquali et al. (contribution 6) highlight how variability in physicochemical parameters impacts the microbial quality and safety of Italian artisanal salami. The authors found that high enterobacteria levels in the meat mixture used for salami elaboration were related to bacterial pathogen occurrence. In addition, suboptimal salami ripening conditions favored the presence of *Staphylococcus aureus* and *L. monocytogenes* in different products and processing environments. Conversely, Wiatrowski et al. (contribution 7) assessed hygiene conditions in food trucks by using various methods including Petrifilm TM and bioluminescence. Swabs and prints from a total of 20 food trucks in Poland were analyzed. The study results highlight the need for detailed hygiene regulations and certified training for food truck personnel to mitigate the risk of bacterial contamination and foodborne infections.

Regarding microbial prevalence and its implications for human health, Wiktorczyk-Kapischke et al. (contribution 8) investigated the presence of *L. monocytogenes* in a salmon processing environment, identifying 38 genetically different strains among 62 isolates, including 6 persistent strains. The authors also identified serogroup 1/2a-3a as the dominant serogroup. Persistent strains showed higher tolerance to disinfectants and higher capacity for biofilm formation. The findings of this study provide information on the phenotypic characteristics of *L. monocytogenes* strains in salmon processing environments. On a related note, Harrison et al. (contribution 9) evaluated potential sources of extraintestinal pathogenic *Escherichia coli* infections using the genomic data of isolates from five U.S. government organizations. Virulence gene analysis of 38,032 isolates categorized into 40 virulence groups revealed associations between sequence types and human disease risk. Medium- and high-risk groups showed a higher prevalence of human-associated sequence types, including ST-131. The food source isolates mostly belonged to low-risk groups, while companion animal isolates predominantly belonged to medium- or high-risk groups. 

This Special Issue also covers the application of predictive modeling to assess the efficacy of inactivation treatments to mitigate the presence of microorganisms in food. Cuggino et al. (contribution 10) collected information on steps, processing parameters, and controls applied in the ready-to-eat leafy vegetable processing industry in Argentina and applied predictive models to estimate *Salmonella* concentrations alongside the production process and distribution chains of fresh-cut lettuce, including the use of chlorine washing as a disinfection method. The findings of their study aid the development of informed risk-based sampling programs and the determination of optimal process parameters for mitigating *Salmonella* spp. in ready-to-eat leafy vegetables. Conversely, González-Tenedor et al. (contribution 11) applied predictive models for assessing the thermal inactivation of *Listeria innocua* in coconut water under isothermal and dynamic conditions, crucial for ensuring product safety in the growing coconut water market. The authors concluded that mild heat treatments offer a viable option for preserving the quality and safety of coconut water but that this form of treatment requires the careful selection of heating conditions to prevent microbial stress adaptation under dynamic conditions.

Pulsrikarn et al. (contribution 12) integrated predictive models into a risk assessment framework to evaluate the health impact of antimicrobial-resistant *Salmonella* spp. in retail pork sold in Thailand. More specifically, the authors assessed the health risks associated with susceptible and quinolone-resistant (QR) *Salmonella* contamination in pork. The probability of illness and mortality rates were estimated for both susceptible and resistant strains, with QR strains showing higher prevalence and lower mean concentrations. Monte Carlo simulations yielded annual mortality rates for QR salmonellosis, aligning with previous reports on the adverse health effects of antimicrobial resistance. Their findings underscore the relevance of addressing antimicrobial resistance in microbial risk assessments. 

Concerning antimicrobial resistance, the authors of the studies included in this Special Issue also examined the prevalence and implications of resistance genes in different contexts. Regecová et al. (contribution 13) investigated antimicrobial resistance and the genes encoding staphylococcal enterotoxins in *Staphylococcus warneri* strains, a pathogen linked to inflammatory diseases in immunosuppressed patients. A total of 45 isolates were obtained from various meat samples and 22% of them displayed multidrug resistance, evidencing the urgent need for effective management strategies. Similarly, Pakbin et al. (contribution 14) explored antibiotic-resistant genes and foodborne pathogens in sweet samples from local markets in Iran. Their study identified *Staphylococcus aureus*, *Cronobacter sakazakii*, *Shigella* spp., *Campylobacter jejuni*, and *Campylobacter coli* at varying degrees of prevalence; *S. aureus* and *Shigella* spp. were noted as being the most prevalent pathogens. Preventive strategies such as the automation of food processing, monitoring the hygiene standards of food handlers, and regular testing for antibiotic resistance are recommended by the authors. 

In a related context, Wang et al. (contribution 15) investigated *Bacillus cereus* prevalence, antimicrobial resistance, and virulence gene profiles in infant formula sourced from supermarkets in Beijing, China. Among 88 isolates recovered from 68 infant formula samples, the prevalence rates in domestic and imported samples were 70.6% and 52.9%, respectively. Most strains carried at least one virulence gene, with similar occurrences of certain genes being noted between domestic and imported brands. Antimicrobial susceptibility analysis showed varied resistance rates, with the rapid growth of *B. cereus* in infant formula prepared at room temperature. The authors highlighted the need for monitoring guidelines to establish accepted levels of *B. cereus* in infant formula.

In conclusion, this Special Issue evidences the collaborative efforts of researchers, policymakers, and industry stakeholders in advancing our understanding of foodborne diseases and implementing practical solutions to enhance food safety worldwide. By addressing novel detection methods, antimicrobial resistance, biopreservation strategies, predictive modeling, and risk assessment, this Special Issue presents advances to reduce the incidence of foodborne diseases. We thank all of the authors for sharing their findings in this Special Issue, as well as the reviewers and editorial team for their hard work in ensuring the high quality of the papers published herein. 
